# Updates on Recent Advancements in Hepatitis D Virus Treatment

**DOI:** 10.3390/v17081100

**Published:** 2025-08-10

**Authors:** Ali Emre Bardak, Nazli Begum Ozturk, Merve Gurakar, Lynette Sequeira, Eda Yildiz, Enis Hikmet Ozmert, Ramazan Idilman, Ahmet Gurakar

**Affiliations:** 1 Department of Medicine, Boston Medical Center-Brighton, Boston, MA 02135, USA; aliemre.bardak@bmc.org; 2 Division of Gastroenterology and Hepatology, Saint Louis University School of Medicine, St. Louis, MO 63104, USA; 3 Division of Gastroenterology and Hepatology, Johns Hopkins University School of Medicine, Baltimore, MD 21205, USA; 4 Department of Surgery, Mayo Clinic, Rochester, MN 55905, USA; 5 Department of Gastroenterology, Ankara University School of Medicine, Ankara 06230, Turkey

**Keywords:** hepatitis D virus, HDV, bulevirtide, lonafarnib, small interfering RNA, antisense oligonucleotide, nucleic acid polymer, interferon lambda

## Abstract

Hepatitis D virus (HDV) infection remains a major cause of severe liver disease among hepatitis B virus (HBV)-infected patients, contributing to accelerated progression to cirrhosis and hepatocellular carcinoma. Pegylated interferon-α remains the first-line therapy for chronic HDV infection in most cases. However, despite its approval for HBV and hepatitis C virus (HCV) infections, its use in HDV is largely driven by a lack of other options and is constrained by its limited efficacy, suboptimal durability of response, and a substantial side effect profile. Meanwhile, bulevirtide, an entry inhibitor, became the first agent to be approved for use in chronic HDV infections by the European Medicines Agency (EMA), and several other therapies are currently being investigated as well. In this review, we provide updates on recent advancements in HDV treatment and novel therapies.

## 1. Introduction

Hepatitis D virus (HDV), also known as hepatitis delta virus, was discovered in the 1970s by Mario Rizzetto [1,2]. It is a defective virus that requires hepatitis B virus (HBV) for its life cycle, making HDV infection possible only in the presence of HBV [1,3]. HDV infection leads to more rapid progression to cirrhosis and higher rates of hepatocellular carcinoma (HCC) than HBV monoinfection [4,5,6]. Chronic HDV infection (CHD) is associated with the most severe and progressive form of viral hepatitis, leading to cirrhosis in 50–60% of cases within 5–10 years and significantly increasing the risk of hepatic decompensation and liver-related mortality [5,7,8]. In this context, the treatment of HDV is of utmost importance. However, therapeutic options remain extremely limited. Currently, no drug approved by the Food and Drug Administration (FDA) is available in the United States for HDV treatment, whereas in Europe, the entry inhibitor bulevirtide has received approval from the European Medicines Agency (EMA) for use in patients with CHD and compensated liver disease [9,10]. Although pegylated interferon alfa (pegIFN-α) has long been used off-label as the primary treatment option, its use is constrained by limited efficacy and a significant side effect profile [11,12,13,14]. Additionally, since HDV relies on the host cell’s RNA polymerase for replication, inhibitors targeting the HBV DNA polymerase are ineffective [14]. Consequently, active efforts to develop new treatments for HDV are ongoing. In this review, we will first provide a general overview of HDV, followed by a discussion of the latest updates in HDV therapies and their clinical implications.

## 2. Epidemiology

HDV prevalence worldwide is estimated to be between 0.8% and 1% [7,15]. However, among individuals positive for the HBV surface antigen (HBsAg), HDV prevalence rises to 5–10%, indicating that roughly 40–60 million people globally may be infected [7,15,16]. Prevalence varies considerably by region: in Africa, rates can reach up to 50% among HBsAg-positive individuals, while in Asia, particularly in high-risk groups in Mongolia and Uzbekistan, rates range from 50% to 82% [5,16]. In contrast, Europe and North America generally report lower overall prevalence, although certain subpopulations or risk groups, such as immigrants, intravenous drug users, patients undergoing regular hemodialysis treatments, men who have sex with men, and sex workers tend to show higher rates [5,13,16].

HDV has eight genotypes, HDV-1 to HDV-8 [17,18]. HDV-1 is the most common globally, particularly in Europe, the Middle East, North America, and northern Africa, while other genotypes show distinct geographic patterns (e.g., HDV-2 in East Asia, HDV-3 in South America’s Amazon Basin, HDV-4 in Taiwan and Japan, and HDV-5 to HDV-8 in Africa) [17,18,19,20].

## 3. Pathogenesis

As a deficient RNA virus, HDV utilizes HBsAg as an envelope, enabling assembly, release, and subsequent entry of the HDV virion into the hepatocytes via the sodium taurocholate cotransporting polypeptide (NTCP) receptor [1,3,9,21,22]. After entering the hepatocyte, the replication occurs in the nucleus via host RNA polymerase II [10,23]. HDV is the smallest known human virus and features a circular, single-stranded RNA genome of 1678 nucleotides [1,2]. This RNA encodes two isoforms of hepatitis D antigen (HDAg): the small HDAg (S-HDAg) and the large HDAg (L-HDAg). S-HDAg primarily facilitates viral replication by interacting with host replication factors and HDV RNA, while L-HDAg is responsible for viral assembly. Both proteins must undergo post-translational modifications to function properly. S-HDAg is primarily modified through phosphorylation, while L-HDAg is isoprenylated, which is crucial for its binding to HBsAg and viral assembly [24,25,26,27]. Furthermore, although HBsAg is required for the HDV life cycle, active HBV replication is not necessary. It is thought that, particularly in chronic cases, the integration of HBV DNA into the host genome is sufficient to maintain the HBsAg levels required for HDV persistence rather than active HBV replication [23].

In addition to its known spread via HBsAg and NTCP, HDV has also been shown to spread independently of both HBsAg and NTCP. The underlying mechanism here is the mitotic division of already infected hepatocytes, leading to the formation of new HDV-infected hepatocytes rather than transmission of the virus to uninfected hepatocytes [28,29,30,31]. The discovery of this mechanism is especially important for the outcome and interpretation of therapies developed to target viral entry.

Immune-mediated liver injury plays a crucial role in the pathogenesis of HDV. The interferon response, primarily triggered via the melanoma differentiation antigen 5 (MDA5) pathway, constitutes the main mechanism [32,33]. This strong interferon-mediated antiviral state can also inhibit HBV replication in addition to hepatocellular injury. Moreover, HDV proteins have been shown to directly interfere with HBV by disrupting RNA synthesis, accelerating RNA decay, and silencing HBV enhancers, further inhibiting HBV replication. For these reasons, HBV replication may be suppressed in HDV infection [33,34,35,36].

## 4. Prognostic Factors

### 4.1. Baseline Liver Injury

Baseline liver injury at the time of HDV diagnosis is a strong predictor of subsequent liver-related morbidity and mortality. A total of 30–70% of HDV-infected patients already have cirrhosis at diagnosis, and this suggests the presence of substantial baseline injury in nearly all cases [9]. Accordingly, it is recommended to assess and monitor the extent of this injury both at diagnosis and during follow-up using noninvasive tools such as FIB-4, APRI, and transient elastography [6,37]. It should be kept in mind that the reliability of transient elastography may be compromised during hepatitis flares because it tends to be higher than actual liver stiffness measurements in the setting of acute inflammation [38].

### 4.2. HDV Genotype

While genotype 1 demonstrates a more aggressive clinical course compared with other genotypes, genotype 5 is associated with a milder trajectory. Two main factors have been proposed to explain genotype 1’s higher replication capacity and greater resistance to interferon therapy [39,40]. Although direct scientific proof is lacking, differences in the geographic distribution of genotypes and the variability in host genetic factors, and thus in the host response to HDV infection, may represent an additional factor underlying the observed differences in clinical course [39].

### 4.3. HBV Status

HDV infection presents mainly in two forms: coinfection (simultaneous acute HBV and HDV infection) and superinfection (HDV infection in background of chronic HBV infection) [4,5]. HBV status at the time of HDV acquisition is the most important prognostic factor for the subsequent course of HDV infection. In coinfection, after an incubation period of about one month, patients typically develop symptoms such as fatigue, loss of appetite, nausea, elevated liver enzymes, and jaundice; this form is usually self-limiting, with only around 2% progressing to chronic infection [4,41]. In contrast, superinfection tends to cause more severe acute hepatitis and have a higher risk of progression to fulminant hepatic failure compared to coinfection. Approximately 80–90% of superinfection cases evolve into chronic hepatitis [4,7,34]. Additionally, when comparing chronic coinfection with superinfection, the latter is associated with a significantly more severe clinical course [7,9,42]. Because cases that progress to CHD are predominantly due to superinfection rather than coinfection, there are unfortunately no clinical studies in the literature directly comparing treatment responses between these two forms. That said, it is not unreasonable to expect a poorer treatment response in superinfection compared with coinfection considering pre-existing liver injury from chronic HBV and already available HBsAg for the utilization of HDV.

In comparison to HBV monoinfection, HDV infection accelerates hepatic injury regardless of its form, resulting in more rapid fibrosis and subsequent progression to decompensated liver disease. HDV rather than HBV is the principal driver of hepatic deterioration in this setting [4,9]. HBsAg positivity serves both as a key marker of therapeutic response for most treatments and an important prognostic factor, since HDV utilizes HBsAg to enter and infect hepatocytes. Accordingly, loss of HBsAg is regarded as a functional cure [5,13]. In contrast, HBV DNA levels are not a reliable prognostic surrogate in CHD, as HDV actively suppresses HBV replication and can utilize HBsAg independently of active HBV replication [23,43,44]. Clinical studies have confirmed that detectable HBV DNA in the presence of ongoing HDV viremia does not meaningfully alter patient outcomes [28,45].

### 4.4. HDV RNA

HDV RNA levels reflect viral load and are used both prognostically and to monitor treatment response. A higher baseline HDV RNA is associated with more advanced liver injury at diagnosis and independently predicts higher future risk of liver-related morbidity and mortality [42,45,46]. Moreover, the clearance or persistence of HDV RNA during therapy is the most important on-treatment prognostic factor. Patients who remain HDV RNA-positive during treatment experience higher rates of liver-related morbidity and mortality than those who achieve viral clearance [39,45,47,48]. Persistent HDV RNA positivity in patients who were noncirrhotic at presentation has been associated with an annual cirrhosis incidence of 4% [48,49]. Accordingly, sustained HDV RNA negativity is a critical endpoint in the management of CHD.

Another important consideration is that current HDV RNA assays exhibit considerable variability in sensitivity and genotype coverage due to methodological differences [28,50]. Therefore, consistently using the same high-sensitive assay to monitor treatment response is advisable.

## 5. Coinfection with HIV

In patients with chronic HBV infection, HIV coinfection is observed in approximately 5%. Among those, 1–20% were observed to have HDV seroconversion, with higher rates reported in regions with higher at-risk populations [15,16,51]. In the US, anti-HDV antibodies were detected in ~4% of HIV/HBV coinfected patients, of whom ~40% had detectable HDV RNA levels [52]. A Switzerland-based study of known HIV-positive patients showed that, among those who were HBsAg-positive, ~15% were anti-HDV-positive and, of these, around 65% had detectable HDV RNA [53]. In an Italian cohort of HBV/HIV coinfected cases, 19% demonstrated HDV seroconversion [54].

HIV infection depletes CD4+ T lymphocytes and impairs their function, which undermines three fundamental antiviral mechanisms: First, CD4+ T lymphocytes are required for the activation and maintenance of CD8+ T lymphocytes that clear infected hepatocytes. Second, they produce IFN-γ, which creates an antiviral environment. Third, they support humoral immune response through B lymphocytes, including the production of anti-HBsAg antibodies. As a result, viral clearance is impaired, leading to a rise in viral load [55,56,57]. Additionally, one might reasonably speculate that HIV-mediated immune suppression could potentially reduce the immune-mediated liver injury that is the main cause of liver injury rather than cytopathy in both HBV and HDV; however, HIV also induces a chronic dysregulated immune state and inflammation, with increased proinflammatory cytokines and nonspecific activation of CD4+, CD8+, and B lymphocytes. This state exacerbates the liver injury even further [58,59,60]. As for antiretroviral therapy, it reduces this immune dysregulation, but does not completely eliminate it [61,62].

Studies have consistently shown that the addition of HDV to HBV/HIV co-infection increases liver-related morbidity and mortality drastically, including acute hepatitis flares, accelerated progression to cirrhosis, and hepatic decompensation. However, there is no consensus on its effect on HCC, with most studies reporting an increase and some finding no significant impact [53,63,64].

In the treatment of HBV/HIV/HDV coinfection, tenofovir plus lamivudine or emtricitabine is used for the HBV/HIV component [13,65]. For the HDV component, treatment options are detailed in the relevant section below.

## 6. Treatment Options

The primary goals of HDV therapy are to achieve both sustained virologic and biochemical responses. A biochemical response is defined by normalization of alanine aminotransferase (ALT) levels. A virologic response is considered to be at least a 2-log decline in HDV RNA, although the ultimate goal is the complete clearance/undetectability of HDV RNA. Early virologic response (EVR) is defined as achieving a virologic response by week 24 of treatment, and sustained virologic response (SVR) is achieving an undetectable HDV RNA level and maintaining this undetectability for 24 weeks after treatment cessation. Achieving EVR is an important predictor of SVR. Another goal is a functional cure: HBsAg loss [5,13]. Achieving these targets is critical to prevent long-term complications such as cirrhosis, hepatic decompensation, and HCC [5,13,66].

**Figure 1:**
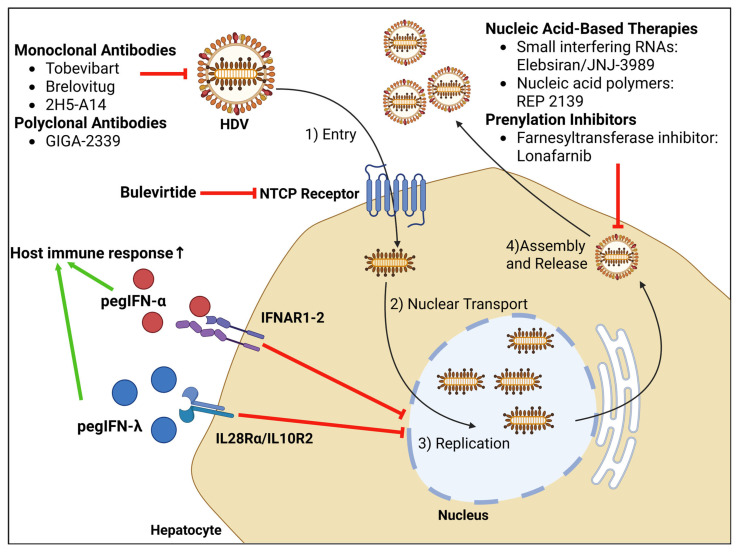
Illustration of the HDV life cycle and sites of action for current and investigational therapies.

Until recently, there was no approved treatment for HDV infection, and the only available option was the off-label use of pegIFN-α. In 2020, with the EMA’s approval of bulevirtide for patients with HDV-associated chronic liver disease, it became the first approved drug for HDV [9,10]. Currently, many drugs are under active development for the treatment of HDV infection (Table 1, Figure 1).

### 6.1. Pegylated Interferon-α

Pegylated interferon-α (pegIFN-α) was originally developed to enhance the efficacy of standard IFN-α for the treatment of chronic HBV and HCV infections, and its use in this context has been approved by the FDA [67,68]. The primary reason for the off-label use of pegIFN-α in CHD is the absence of any approved treatment [10,13]. The underlying rationale for using pegIFN-α in HDV infection was to harness its antiviral and immunomodulatory properties. Through these mechanisms, pegIFN-α inhibits viral replication in infected hepatocytes for both HBV and HDV and facilitates the clearance of infected cells by triggering the host immune response [13,69,70]. Although pegIFN-α demonstrates some efficacy in treating HDV infection, its limited effectiveness and adverse side effects have prevented its approval for this indication [5,71,72].

PegIFN-α is available in two formulations for treatment, pegIFN-α-2a and pegIFN-α-2b, with dosing regimens of 180 mcg/week and 1.5 mcg/kg/week, respectively [12,13]. The recommended treatment duration is 48 weeks. If EVR is not achieved, the likelihood of an SVR is less than 5% [5,13]. SVR rates have been reported as 23–57% in different studies [13]. In the HIDIT-1 study, SVR was observed in 40% of patients, with 12% maintaining this response over a median follow-up of 4.3 years [73,74]. In the HIDIT-2 study, the treatment duration was extended to 96 weeks. Although a higher HDV RNA clearance rate was achieved at week 96, no significant difference in post-treatment SVR was found [12]. Combination therapy of pegIFN-α with HBV-suppressing nucleos(t)ide analogues (NA) has not significantly improved HDV virologic responses compared to pegIFN-α alone [12,74,75]. Finally, pegIFN-α is contraindicated in patients with decompensated cirrhosis [13].

### 6.2. Bulevirtide

Bulevirtide is currently the only antiviral drug approved for the treatment of HDV. In 2020, the EMA approved its use at a dose of 2 mg per day for patients with CHD and compensated liver disease, and studies are ongoing to evaluate its efficacy at higher doses in patients with decompensated liver disease [9,10,71].

Bulevirtide is a lipopeptide that inhibits the NTCP receptors and thereby blocking HBV and HDV from entering and infecting hepatocytes [10,21,76]. These receptors are predominantly expressed on hepatocytes and are primarily responsible for the enterohepatic circulation of bile acids by facilitating the sodium-dependent uptake of conjugated bile acids from the portal system into the liver [77,78,79]. When NTCP function is impaired, the enterohepatic circulation of bile acids is disrupted, leading to increased serum levels of conjugated bile acids, creating a condition known as hypercholanemia [80,81]. This disruption can sometimes indirectly affect the bilirubin metabolism and progress to cholestasis [82,83].

Both HBV and HDV enter hepatocytes using the envelope protein HBsAg [21,84]. HBsAg is composed of small, middle, and large surface proteins encoded by the S gene of the HBV genome [85,86]. The large surface protein contains the preS1 region, which is crucial for the viral entry process [21,84,85]. Initially, HBsAg binds with low affinity to heparan sulfate proteoglycans on the hepatocyte surface [84,87,88]. This is followed by high-affinity binding between the preS1 domain and NTCP, resulting in the internalization of the virus–receptor complex and viral entry into the cell [21,84,89]. Bulevirtide binds to the NTCP receptor, blocking the preS1 domain’s interaction with the receptor and thereby inhibiting viral entry [71,76].

Bulevirtide was developed by MYR GmbH in Germany, and its use was investigated in MYR trials. In the MYR301 phase 3 trial, 150 patients with CHD (excluding those with decompensated cirrhosis) were randomized into three groups: one group received bulevirtide at a dose of 2 mg daily for 144 weeks, a second group received 10 mg daily for 144 weeks, and a third group received no treatment for the first 48 weeks followed by 10 mg daily for the subsequent 96 weeks. At the 48-week evaluation, a combined virologic and biochemical response was observed in 2% of patients in the delayed treatment group, 45% in the 2 mg group, and 48% in the 10 mg group. When analyzed separately, both virologic and biochemical responses appeared dose-independent at week 48 [71]. By week 96, there was no significant difference between the 2 mg and 10 mg groups, with combined response rates of 55% and 56%, respectively. Similarly, no significant differences were observed between the groups when virologic and biochemical responses were evaluated individually [90]. This trend continued at week 144, with combined response rates of 57% in the 2 mg group and 54% in the 10 mg group [91]. As for HDV clearance, HDV RNA clearance was achieved in 0% of the delayed treatment group, 12% of the 2 mg group, and 20% of the 10 mg group at week 48. It continued to increase throughout treatment, reaching 20% (2 mg group) and 36% (10 mg group) by week 96, and further rising to 29% (2 mg group) and 50% (10 mg group) by week 144 [71,90,91]. Notably, 43% of the nonresponders and 82% of the partial responders identified at the week 24 evaluation achieved a virologic response by week 96 [90,91]. Another notable result was that the use of NA did not have a significant impact on the virologic response [71,90]. Drug resistance was assessed following a 24-week treatment period, combining data from MYR202 and MYR301. No resistance was identified by week 24 in any patient [92].

According to the recently presented 48-week post-treatment follow-up data from 64 patients, sustained HDV RNA clearance was observed in 36% patients overall, and specifically in 50% of the 2 mg group, 40% of the 10 mg group, and 23% of the delayed treatment group. Sustained clearance was associated with lower baseline viral load (<4.5 log IU/mL) and lower baseline HBsAg levels. Additionally, the earlier the clearance was achieved before the end of treatment, the more likely it was to be sustained. HBsAg loss or a decrease in ≥1 log IU/mL, as well as the development of antidrug antibodies by week 144, were similarly associated with sustained HDV clearance [93].

In a pooled analysis of paired liver biopsies from MYR202, MYR203, and MYR301, the intrahepatic impact of bulevirtide was assessed by comparing liver biopsy samples obtained at baseline with those obtained after 24 or 48 weeks of treatment. A dose-dependent reduction in intrahepatic HDV RNA levels was demonstrated at week 24, with a median reduction of 0.9 log IU/mL in the 2 mg/day group, 1.1 log IU/mL in the 5 mg/day group, and 1.4 IU/mL in the 10 mg/day group. This downward trend persisted, and, at week 48, HDV RNA reduction was 2.2 log IU/mL in the 2 mg group and 2.7 log IU/mL in the 10 mg group compared to baseline. In contrast to the assessment at week 24, the reduction in HDV RNA at week 48 was dose-independent. This reduction in viral load was paralleled by a significant decrease in the number of HDAg-positive hepatocytes, with approximately 50% of patients showing undetectable levels at week 48. Additionally, the reduction in intrahepatic HDV RNA was correlated with reductions in serum HDV RNA levels and the expression of inflammatory chemokines and interferon-stimulated genes [94].

In the European SAVE-D trial, 244 patients with HDV-related cirrhosis received bulevirtide 2 mg daily for 96 weeks. A total of 95% of the participants had compensated cirrhosis. The study demonstrated significant virologic and biochemical responses. A total of 53% of patients reached a virologic response at week 24, which increased to 65% at week 48 (with 28% achieving undetectable levels), 67% at week 72 (38% undetectable), and 79% by week 96 (48% undetectable). Correspondingly, median HDV RNA levels dropped significantly by 3 log IU/mL at week 96. Biochemical response was achieved in 54% by week 24, 61% at week 48, 61% at week 72, and 64% by week 96. Finally, the combined response was 33% at week 24, which improved to 44% at week 48, 46% at week 72, and 54% at week 96 [95]. In a retrospective analysis of 48 cirrhotic patients who reached week 144 of treatment in the continuation of the SAVE-D trial, 75% achieved a virologic response (with 35% reaching undetectable levels), 67% achieved a biochemical response, and 56% achieved a combined response. Liver stiffness measurements also decreased significantly [96].

In the ongoing multicenter Italian D-SHIELD study, 404 patients with CHD (75% with cirrhosis) started daily 2 mg bulevirtide monotherapy. At baseline, cirrhotic patients were older, had more portal hypertensive complications, and slightly lower HDV RNA levels compared to noncirrhotic patients. According to data at week 48 of treatment, when comparing cirrhotic and noncirrhotic groups, virologic, biochemical, and combined response rates were 66% vs. 65% (*p* = 1), 73% vs. 55% (*p* = 0.06), and 54% vs. 33% (*p* = 0.05), respectively. The extent of HDV RNA reduction and clearance was also found to be similar between the groups [97]. Additionally, during the 3 year follow-up period to date, liver decompensation was observed in eight patients (3.3%), all of whom had clinically significant portal hypertension at baseline [98].

A retrospective case series investigated the off-label use of bulevirtide 2 mg daily in 19 patients with CHD and decompensated Child–Pugh B cirrhosis for a median duration of 41 weeks. A total of 74% (14/19) of patients reached a virologic response after a median of 17 weeks, while 16% (3/19) had a partial response and 11% (2/19) showed no response. Notably, four patients with initial virologic response experienced a relapse with an HDV RNA increase in over 1 log. Subsequently, HDV RNA levels declined in two of these patients and remained elevated in one patient. The relapse in the fourth patient was detected at the end of the observational period, with no further follow-up data available. A total of 74% (14/19) of patients had a biochemical response after a median of 13 weeks, and a combined response was achieved in 42% (8/19) at a median of 19 weeks. Clinically, downstaging from Child–Pugh B to A was observed in 47% (9/19) of the patients. Among the 12 patients with ascites at baseline, 58% (7/12) showed improvement, enabling a reduction or discontinuation of diuretic therapy. Although individual patients exhibited both improvement and deterioration, the overall median Child–Pugh and MELD scores remained stable [99].

Bulevirtide has demonstrated an overall favorable safety profile. Across clinical studies, the most frequently observed adverse events include increases in serum bile acid levels, injection-site reactions, headache, and pruritus. The increase in serum bile acid levels is linked to the inhibition of the NTCP receptor, which disrupts normal bile acid homeostasis. Although these increases were common, they rarely translated into clinically significant symptoms [95,100]. In the MYR301 study, both the 2 mg and 10 mg dosing regimens showed similar rates of adverse events. The extension of treatment did not lead to an increase in serious adverse events, and there were no discontinuations related to bulevirtide-associated toxicity [71,90,100].

The risk of relapse is high when the treatment is discontinued. In the MYR202 phase 2 trial, patients with CHD were treated with bulevirtide (2 mg, 5 mg, or 10 mg daily). HDV RNA levels in patients who discontinued the treatment at week 24 rebounded significantly, with median increases in 1.923 log IU/mL in the 2 mg group, 1.732 log IU/mL in the 5 mg group, and 2.030 log IU/mL in the 10 mg group from week 24 to week 48 [101]. Similar results were obtained in the MYR301 phase 3 trial [71,90]. In the SAVE-D trial, three patients stopped bulevirtide at week 48 after maintaining 24 weeks of undetectable HDV RNA. Of these, two remained undetectable at the follow-up visits 88 and 116 weeks after stopping, while one experienced a relapse 43 weeks after discontinuation [95]. Based on these data, long-term and continuous use of bulevirtide is recommended to maintain virological suppression [71,90,95,101,102].

### 6.3. Combination Therapy of Bulevirtide and PegIFN-α

In the MYR204 phase 2 trial, the efficacy of the combination therapy of bulevirtide and pegIFN-α was assessed. A total of 175 patients with CHD and elevated but less than 10 times of upper limit of normal ALT levels, were enrolled, and were randomized into four different treatment groups, including pegIFN-α-2a 180 mcg weekly monotherapy for 48 weeks, bulevirtide 2 mg daily plus pegIFN-α-2a 180 mcg weekly for the first 48 weeks of a 96-week bulevirtide course, bulevirtide 10 mg daily plus pegIFN-α-2a 180 mcg weekly on the same schedule, and bulevirtide 10 mg daily monotherapy for 96 weeks. Nearly one third of patients in each group had cirrhosis, none of which was decompensated. All patients were followed for 48 weeks post-treatment. End-of-treatment undetectable HDV RNA rates were 21% (week 48), 44% (week 96), 70% (week 96), and 22% (week 96) in the respective groups. At 24 weeks post-treatment, HDV RNA was undetectable in 17%, 32%, 46%, and 12%. The difference in terms of undetectable HDV RNA rates at 24 weeks post-treatment between the 10 mg bulevirtide plus pegIFN-α-2a group and the 10 mg bulevirtide monotherapy group was found statistically significant. At 48 weeks post-treatment, undetectable HDV RNA rates remained 25%, 26%, 46%, and 12%, respectively. The rates of the composite outcome of undetectable HDV RNA and biochemical response at 48 weeks post-treatment were 25%, 22%, 40%, and 8%, respectively. In conclusion, this study indicated that the use of pegIFN-α in combination with bulevirtide was more effective than bulevirtide monotherapy, particularly in terms of virologic response [10].

Following the encouraging results of the MYR-204 phase 2 trial, the Swedish SEE-D observational study was launched. CHD patients with advanced fibrosis or compensated cirrhosis were planned to be treated with bulevirtide 2 mg daily plus pegIFN-α-2a for 96 weeks (180 mcg in the first 48 weeks, then 135 mcg). In the ongoing study, interim analyses show that all patients assessed at week 24 (19 patients) and week 48 (29 patients) achieved a virologic response, with HDV RNA becoming undetectable in 34.5% and 78.9%, respectively. These early results reinforce the potential benefit of combining pegIFN-α with bulevirtide [103].

In this pooled analysis of MYR204 and MYR301, CHD patients treated with bulevirtide 10 mg daily—alone or with pegIFN-α—for at least two years were evaluated. Sustained HDV suppression at 48 weeks post-treatment was significantly more likely in those with undetectable HDV RNA at end of treatment, while those with residual viremia frequently relapsed. Combination therapy and longer treatment duration provided better outcomes [104].

A mathematical model was developed to determine the minimum duration of HDV viral load monitoring required during bulevirtide treatment to reliably predict long-term response. Data for the development of this model were obtained from 414 patients enrolled in the MYR202, MYR203, MYR204, and MYR301 studies. The model showed that monitoring HDV viral load during the first 48 weeks accurately (>90%) predicts treatment response at 96 weeks, with minimal additional benefit from extended monitoring beyond that point. The analysis also demonstrated that bulevirtide 10 mg is more effective than 2 mg, and that adding pegIFN-α further improved HDV clearance [105].

The therapeutic predictive value of HBsAg composition based on the ratios of small, middle, and large surface proteins was analyzed in a cohort of 106 patients treated with either 180 mcg weekly pegIFN-α (56 patients) or 2 mg daily bulevirtide (50 patients). Regardless of treatment, baseline ratios of middle and large surface proteins were significantly lower in patients who achieved HBsAg loss, virologic response, or undetectable HDV RNA compared to nonresponders. Patients in whom large surface protein made up <4.8% of total HBsAg were more likely to achieve a virologic response and undetectable HDV RNA. A decrease in the ratio of middle surface protein was observed during treatment in both treatment groups among those who achieved undetectable HDV RNA, while no such change was seen in nonresponders. Furthermore, patients with a virologic response after bulevirtide treatment had lower post-treatment levels of both middle and large surface proteins compared to nonresponders [106].

### 6.4. Lonafarnib

Lonafarnib is an orally administered farnesyltransferase inhibitor. Initially developed for cancer therapy, it was later approved by the FDA in 2020 for use in Hutchinson–Gilford Progeria Syndrome and processing-deficient progeroid laminopathies [107,108]. Farnesyltransferase is also the enzyme that mediates the prenylation of L-HDAg and is, therefore, directly involved in the viral assembly of HDV. Inhibition of this step prevents the formation and release of new HDV particles, aiming to confine the virus within the cell and thereby limit viremia [24,26,72,109]. Lonafarnib’s potential efficacy in treating HDV infection was first demonstrated in animal models; subsequently, human studies were initiated [110,111,112].

The first in-human proof-of-concept study, which enrolled 14 patients with HDV-associated compensated liver disease, was published in 2015. Six patients received 100 mg of lonafarnib twice daily, six received 200 mg twice daily, and two received a placebo for 28 days. At the end of the 28-day treatment period, both treatment groups showed a significant mean reduction in log HDV RNA levels, 0.73 log IU/mL in the 100 mg twice daily group, and 1.54 log IU/mL in the 200 mg twice daily group. Serum lonafarnib concentrations were correlated with reductions in HDV RNA levels (r^2^ = 0.78, *p* < 0.0001). The majority of patients experienced gastrointestinal side effects such as nausea, diarrhea, and abdominal bloating; however, none of these led to treatment discontinuation [113].

Another proof-of-concept study was LOWR HDV-1 in 2018. Patients were divided into five groups of three patients: the first group received lonafarnib 200 mg twice daily for 12 weeks, the second received 300 mg twice daily for 12 weeks, the third received 100 mg three times daily for 5 weeks (due to drug supply limitations), while the fourth and fifth groups received combination therapies with either pegIFN-α 180 mcg once weekly for 8 weeks or ritonavir 100 mg once daily added to lonafarnib 100 mg twice daily for 8 weeks. When used as monotherapy, lonafarnib resulted in a dose-dependent reduction in HDV RNA levels, with greater decreases observed at higher doses. On the other hand, gastrointestinal side effects were notably more severe at higher doses. The groups receiving ritonavir and pegIFN-α alongside lonafarnib demonstrated a greater reduction in HDV RNA despite using a lower dose of lonafarnib and also a more favorable gastrointestinal side effect profile compared to the lonafarnib monotherapy groups. It was concluded that using ritonavir, a CYP3A4 inhibitor, results in increased bioavailability and fewer side effects [114].

The subsequent continuation study of LOWR HDV-1 was LOWR-2. A total of 55 patients with HDV-associated compensated liver disease were enrolled. The study aimed to determine the optimal drug dose and combination regimens. Participants were allocated into three primary groups based on their regimen: one group received a high-dose lonafarnib (75, 100, or 150 mg twice daily) combined with ritonavir regimen for 12 weeks; a second group received a low-dose lonafarnib (25 or 50 mg twice daily) combined with ritonavir regimen for 24 weeks; and a third group was treated with a triple combination regimen consisting of a low-dose lonafarnib (25 or 50 mg twice daily) in addition to ritonavir and pegIFN-α (180 mcg weekly) for 24 weeks. The primary endpoint was achieving a virologic response. The low-dose lonafarnib with ritonavir regimen demonstrated comparable efficacy with fewer side effects than the high-dose lonafarnib with ritonavir regimen. A virologic response was observed in 46% (6/13) of patients treated with lonafarnib 50 mg twice daily and ritonavir, compared to 89% (8/9) of patients receiving a triple combination regimen. As for gastrointestinal side effects, they were markedly lower in the low-dose lonafarnib regimens compared to high-dose lonafarnib regimens [109].

Finally, in the D-LIVR study, 407 patients with CHD-associated compensated liver disease receiving NA were randomized into four groups: lonafarnib 50 mg twice daily with ritonavir, lonafarnib 50 mg twice daily with ritonavir and pegIFN-α, pegIFN-α, or a placebo for 48 weeks. A paired liver biopsy was obtained at week 48. At the end of the treatment, virologic response rates were 14.6% in the lonafarnib plus ritonavir group, 32% in the lonafarnib plus ritonavir plus pegIFN-α group, 36.5% in the pegIFN-α monotherapy group, and only 3.8% in the placebo group. Biochemical response was observed in 24.7%, 34.4%, 11.5%, and 7.7% of patients, while combined response rates were 10.1%, 19.2%, 9.6%, and 1.9% in the respective groups. The rates of patients who achieved an improvement of more than two points on the histology activity index without any worsening of fibrosis were 33%, 53%, 38%, and 27%, respectively. Overall, it was reported that lonafarnib was well tolerated and that the groups were comparable in terms of side effects [115].

### 6.5. Pegylated Interferon-λ (PegIFN-λ)

Pegylated interferon-λ (pegIFN-λ) is a type III interferon, which acts through a receptor complex consisting of IL-28Rα and IL-10R2 subunits [116,117]. This receptor complex is distinct from the receptors of type I (IFN-α and IFN-β) and type II (IFN-γ) interferons, and is more selectively expressed on epithelial cells, including hepatocytes [116,117,118,119,120]. Thus, compared with type I and type II interferons, IFN-λ is associated with a more targeted immune response and more favorable safety profile, including fewer systemic side effects [72,116,121,122].

Clinical studies have shown that IFN-λ exhibited comparable treatment outcomes to IFN-α in HBV and HCV infections, while offering a more favorable side-effect profile [123,124,125]. Investigations into its application for HDV are ongoing.

In the phase 2 LIMT-1 trial, 33 patients with CHD and compensated liver disease received weekly pegIFN-λ at either 120 mcg or 180 mcg weekly for 48 weeks, followed by 24 weeks of follow-up. At week 72, the virologic response rate was 16% (3/19) in the 120 mcg group versus 36% (5/14) in the 180 mcg group. As for the combined virologic and biochemical response rates at this time point, they were 11% and 29% in the 120 mcg and 180 mcg groups, respectively. Treatment was discontinued due to hyperbilirubinemia in eight patients: 4/19 in the 120 mcg group and 4/14 in the 180 mcg group [126].

In the phase 3 LIMT-2 trial involving patients with CHD and compensated liver disease, hepatobiliary events causing liver decompensation occurred in four participants, prompting early termination of the trial and suspension of treatment for all patients [127].

### 6.6. Nucleic Acid-Based Therapies

Nucleic acid-based therapies include small interfering RNAs (siRNAs), antisense oligonucleotides (ASOs), and nucleic acid polymers (NAPs) [128]. SiRNAs and ASOs are therapeutic agents designed to silence specific genes by targeting RNA molecules, and thereby, they aim to reduce the production of HBV-associated viral proteins, including HBsAg [129,130]. NAPs interfere with the assembly and secretion of the HBV-associated viral proteins, including HBsAg [128,131].

In general, siRNAs are double-stranded RNA fragments that become incorporated into the RNA-induced silencing complex (RISC). The RISC uses one strand of the siRNA as a guide to recognize and bind to complementary sequences on mRNA or viral RNA, leading to their cleavage and degradation and thereby preventing protein synthesis [132,133]. ASOs are single-stranded DNA sequences that bind directly to their target RNA through complementary base pairing. This leads to either degradation of the target RNA (e.g., mRNA) by activating the RNase H enzyme or blocking the translation process [129,130]. NAPs are single-stranded phosphorothioated oligonucleotides that act through binding to membranes and proteins involved in the assembly and secretion of viral particles [128,131].

Most nucleic acid-based therapies are in early stages and have generally been developed as HBV therapies that may also apply to HDV [134,135,136].

Elebsiran (VIR-2218), a siRNA designed to degrade HBV mRNA and reduce expression of HBV proteins, including HBsAg, was evaluated both as monotherapy and in combination with pegIFN-α-2a, achieving dose-dependent declines in HBsAg levels. Of note, no patient receiving elebsiran alone achieved HBsAg clearance [137]. Following these results, elebsiran was administered in combination with tobevibart (VIR-3434), a monoclonal antibody against HBsAg, for CHD as part of the phase 2 SOLSTICE trial. With this dual regimen, all patients achieved a virologic response by week 24. HDV RNA became undetectable in 41% of patients at week 24 and 64% at week 36. Additionally, in a subgroup treated through week 60, the rate of undetectable HDV RNA rose to approximately 80% [138]. Vir Biotechnology announced that phase 3 of the study is scheduled to commence in 2025.

JNJ-3989 (JNJ-73763989) is an siRNA developed to inhibit the transcription of all HBV RNA including mRNA and pgRNA and translation of HBV viral proteins. Its use in CHD was evaluated in the REEF-D proof-of-concept study. In the first part of this trial, 17 patients received JNJ-3989 in combination with a NA; four patients achieved a combined virologic and biochemical response by week 48. Treatment was discontinued early in eight patients due to the absence of any HBsAg decline and ALT elevation, which was observed in patients with relatively high baseline HBsAg (>10,000 IU/mL) and HDV RNA levels (HDV RNA > 100,000 IU/mL) [139,140]. Following these results, the second part of the study was initiated, excluding patients with cirrhosis or having both HBsAg > 10,000 IU/mL and HDV RNA > 100,000 IU/mL at their baseline. The planned duration for the trial is determined to be 144 weeks, and the interim results at week 48 were announced. 7/15 (47%) patients on JNJ-3989, had virologic response while also maintaining normal ALT levels. 7/24 (29%) patients had ALT elevations, six of whom had a baseline HBsAg > 10,000 IU/mL. Pooled data from Parts 1 and 2 at week 48 showed that 14/27 patients achieved a virologic response, 11 of whom also had the biochemical response. Notably, all patients with baseline HBsAg levels > 10,000 IU/mL experienced ALT elevations during treatment [141].

No ASOs has been evaluated in human clinical studies for HDV infection to date. Bepirovirsen (GSK3228836) is an ASO against HBV mRNAs, and one of the most promising agents in its class. It achieved sustained HBsAg and HBV DNA loss in 9–10% of participants by week 24, regardless of concomitant NA use. Thereby, bepirovirsen holds potential for use in the treatment of HDV infection [142,143].

REP 2139 is a NAP, which was used for the treatment of HBV and HDV. It demonstrates its effect primarily by binding to host chaperone proteins and inhibiting the assembly and secretion of HBsAg [144,145,146]. In the Phase 2 REP 301, twelve noncirrhotic, treatment-naive patients with chronic HBV/HDV coinfection, each HBeAg-negative and with HBsAg levels >1000 IU/mL, were enrolled to receive REP 2139-Ca therapy. Patients were given REP 2139-Ca 500 mg intravenously weekly for 15 weeks, then REP 2139-Ca 250 mg plus pegIFN-α-2a 180 mcg weekly for 15 weeks, and, finally, pegIFN-α-2a 180 mcg weekly alone for 33 weeks. At the end of therapy, half of the patients achieved HBsAg negativity, and five of these remained HBsAg-negative at one-year follow-up. Anti-HBs seroconversion occurred in five patients and persisted through the one-year follow-up. During treatment, HDV RNA became undetectable in 11 patients, and seven maintained HDV RNA negativity at one-year follow-up [145]. REP 301-LTF is the long-term follow-up study of REP 301, where 11 patients were monitored for 3.5 years after completing the treatment. Seven patients maintained undetectable HDV RNA levels and biochemical response. Of these, four had undetectable HBV DNA and HBsAg loss, while three had HBV virologic control with HBV DNA ≤ 2000 IU/mL [147].

Finally, in 2024, under the Replicor Compassionate Access Program (RCAP), 33 patients with HBV/HDV coinfection and cirrhosis were enrolled to receive REP 2139-Mg, which is the subcutaneous form of REP 2139-Ca with the same pharmacokinetics. Each patient had virologic or biochemical failure or rebound during prior pegIFN-α (24/33) and/or bulevirtide (20/33) therapy. Over a 48-week course, all patients received REP 2139-Mg 250 mg weekly plus daily tenofovir disoproxil fumarate (28/33) or alafenamide (5/33). 18 patients without decompensated cirrhosis and contraindications against pegIFN-α, also received pegIFN-α-2a (45–180 mcg/week). By the end of therapy, 76% (25/33) achieved virologic response for HDV, and 61% (20/33) had undetectable HDV RNA levels. Among the 19 patients with post-treatment follow-up (4 to 48 weeks), these rates were 79% (15/19) and 63% (12/19), respectively. For HBsAg, 52% (17/33) reduced levels below 10 IU/mL, 24% (8/33) lost HBsAg, and 30% (10/33) seroconverted to anti-HBs by end of therapy, with corresponding follow-up rates of 47% (9/19), 25% (5/19), and 42% (8/19). No statistically significant differences were observed in HDV virologic outcomes between patients treated with pegIFN-α and those who were not. In the pegIFN-α group, 78% (14/18) achieved a virologic response versus 73% (11/15) in the non-pegIFN-α group, and 67% (12/18) of pegIFN-α-treated patients had undetectable HDV RNA compared with 53% (8/15) of those without pegIFN-α [148].

### 6.7. Monoclonal Antibodies

The development of monoclonal antibodies for both HBV and HDV is ongoing; however, no agent has completed phase 3 trials yet. The most advanced candidates are tobevibart (VIR-3434) and brelovitug (BJT-778).

Tobevibart is a monoclonal antibody directed against the antigenic loop of HBsAg. In preclinical studies, it demonstrated activity against all HBV genotypes and was found to be more potent than hepatitis B immunoglobulin (HBIG) for both HBV and HDV [149]. Clinically, tobevibart has been evaluated both as a monotherapy (tobevibart 300 mg every 2 weeks) and in combination with the siRNA elebsiran (VIR-2218) (tobevibart 300 mg plus elebsiran 200 mg every 4 weeks) in the phase 2 SOLSTICE trial. Notably, combination therapy clearly outperformed tobevibart monotherapy, particularly in terms of virologic response. As detailed above, all patients in the combination group achieved a virologic response by week 24, and the rates of HDV RNA undetectability were also highly promising, showing a steady increase with longer treatment duration. In the combination therapy group, baseline cirrhotic status, HDV RNA levels, and HBsAg levels were not found to have any predictive value for achieving HDV RNA undetectability. However, in the tobevibart monotherapy group, higher baseline HDV RNA levels were inversely correlated with virologic response. The phase 3 of the study was announced to commence in 2025 [138,150].

Similar to tobevibart, brelovitug (BJT-778) is also targeting HBsAg, and is currently being evaluated for CHD in the ongoing phase 2 trial, which is set to assess three different dosing regimens for 48 weeks in three groups as follows: 300 mg weekly, 600 mg weekly for 12 weeks followed by 600 mg every 2 weeks, and 900 mg every 2 weeks for 4 weeks followed by 900 mg every 4 weeks. Due to limited efficacy, data from Group 3 were not disclosed. Reported results included data from Group 1 at weeks 12 and 28, and from Group 2 at week 12, allowing a comparison between 300 mg and 600 mg weekly dosing. In Group 1, by week 12, 7/10 patients achieved a virologic response, 5/9 had a combined response, and only 1/10 patient had undetectable HDV RNA. By week 28, all 10 patients achieved a virologic response, 6/9 had a combined response, and 6/10 had undetectable HDV RNA. In Group 2, at week 12, 8/10 patients achieved a virologic response, 2/4 achieved a combined response, and 3/10 had undetectable HDV RNA. Brelovitug was designated as a Breakthrough Therapy for CHD by the FDA, marking it as a high-potential therapy for the disease. Additionally, it also received both Priority Medicines and Orphan Drug designations from the EMA. As of March 2025, the phase 2b/3 AZURE-1 trial for brelovitug has also begun enrolling patients [151,152,153].

RG-6449 (RO7565020) is a monoclonal antibody that targets the antigenic loop of HBsAg. It has shown encouraging results in lowering HBsAg levels in preclinical studies and an ongoing phase 1 trial. However, it has not yet been studied for the treatment of CHD [154,155].

Other monoclonal antibodies in development include 2H5-A14, E6F6, N6HB426-20, and CIM212930. 2H5-A14 functions by blocking the preS1 and NTCP interaction, and was shown to successfully reduce both serum HBsAg and intrahepatic cccDNA levels in mouse models [156]. As another promising monoclonal antibody, E6F6 suppresses both HBsAg and HBV DNA by facilitating Fcγ receptor-dependent phagocytosis of HBsAg. Additionally, it is also reported to restore the T lymphocyte response to HBV, and thus potentially help with immune reconstitution in chronic HBV infection [157]. N6HB426-20 is an agent that directly targets NTCP and shows promising results in in vivo human hepatocytes and in vitro mouse models [158]. CIM212930 is another NTCP-targeting agent that is also being developed for use in cholestatic liver disease, having shown improvement in cholestatic liver injury in preclinical animal models [159].

### 6.8. Polyclonal Antibodies

GIGA-2339 is a first-in-class, fully human recombinant polyclonal antibody that contains >1000 distinct IgG clones directed against HBsAg. Since the antibody pool recognizes multiple epitopes, it is intended to remain active across all HBV genotypes and against both pre-existing and treatment-emergent HBsAg variants, thereby achieving a functional cure. In preclinical studies and an ongoing phase 1 trial, its neutralizing potency exceeded plasma-derived HBIG by >2000-fold. It also stimulated antibody-dependent cellular cytotoxicity and antibody-dependent cellular phagocytosis about 10-fold more effectively than HBIG. Another notable finding was that GIGA-2339 was able to neutralize HDV-1 particles coated with HBsAg from all eight HBV genotypes (A–H). These findings suggest that GIGA-2339 has the potential to be used not only in the treatment of HBV but also in the treatment of HDV in the future [160,161].

## 7. Liver Transplantation

CHD often progresses to end-stage liver disease, with an estimated 30–70% of patients already cirrhotic at diagnosis and cirrhosis developing annually approximately in 4% of the noncirrhotic patients [9,48,49]. Additionally, the risk of developing HCC is approximately doubled compared with HBV monoinfection. Consequently, 10-year survival rates are below 50% primarily due to liver-related conditions [4,9]. When HDV infection leads to end-stage liver disease, HCC, or fulminant hepatitis, liver transplantation remains the only viable option, and considering the relatively fast disease progression, timely referral for transplantation is critical [162,163]. Notably, patients with HDV-related cirrhosis are younger at transplant listing compared to patients with HBV monoinfection-related cirrhosis, and the indication for transplantation is predominantly liver decompensation, with HCC accounting for a smaller proportion [164]. Patients with CHD also tend to experience more acute decompensation compared to those with HBV monoinfection [162,164]. 

Management of the pretransplant period does not differ significantly from CHD management. NA are preferred to suppress active HBV replication, while HDV therapy is continued as described in the relevant section above [13,165,166]. To date, no comparative studies have evaluated the impact of the chosen HDV treatment modality during the pretransplant period on transplantation outcomes.

Recurrence is the key post-transplant concern. The standard prophylactic regimen to prevent recurrence of HDV infection is the lifelong use of NA combined with HBIG, while HDV-specific suppression therapy is not, at least for now, part of this standard approach [162,167,168,169]. Adoption of this protocol has reduced post-transplant recurrence rates to ~10% for HBV and below 5% for HDV. Aside from recurrence, it also produced substantial improvements in other long-term outcomes, such as graft dysfunction, rejection, and survival rates, which do not differ from HBV monoinfected recipients, with 5-year survival exceeding 80% now [162,167,169,170,171,172,173].

Although lifelong combination therapy with NA and HBIG is recommended, recent studies demonstrate that discontinuing HBIG after 6–24 months still maintains comparable recurrence rates with combination therapy [174,175,176,177,178]. While further research is needed, current evidence supports considering this strategy in selected cases.

Moreover, there is currently a gap in evidence regarding the use of newer therapies such as bulevirtide in the post-transplant setting, and future studies aimed at addressing this gap can be anticipated.

De novo HDV infection after liver transplantation is uncommon but still a clinical concern [168,173]. Risk minimization measures include avoiding grafts from anti-HDV-positive donors; treating HBsAg-positive recipients with NA plus HBIG, with dose and duration adjusted based on individual risk; and administering NA therapy to recipients of grafts from HBsAg- or anti-HBc-positive donors, adding HBIG for high-risk patients and using brief or no HBIG in low-risk cases [13,162,168,179]. Additionally, all liver transplant candidates should be vaccinated for HBV as indicated [180].

## 8. Conclusions

The introduction of new therapies, most notably entry inhibitor bulevirtide, changed the therapeutic landscape for chronic HDV infection. However, the durability of response to new treatment options, the optimal combination regimens, and their long-term safety profiles require further investigation in ongoing and future studies.

## Figures and Tables

**Table 1:** Summary of current and investigational therapeutic agents for HDV infection.

Therapeutic Classes	Agent and Regimen(s)	Route	Key Antiviral Mechanism	Development/Regulatory Status	Typical Study Regimens ^†^	Notes
Interferons	PegIFN-α-2a/-2b	SC	Broad immune activation	Approved for HBV/HCV → off-label HDV	180 mcg qwk/1.5 mcg/kg qwk × 48 wk	SVR 23–57%; if no SVR → EVR < 5%; limited durability; contraindicated in decompensated cirrhosis; limited use due to side effects
	PegIFN-λ	SC	Type III IFN, hepatocyte-selective	Phase 2/3 (LIMT-2 Phase 3 trial terminated due to liver decompensation in participants)	120–180 mcg qwk × 48 wk	CR ≤ 30%; trial halted due to liver decompensation secondary to hepatobiliary toxicity
Viral entry inhibition	Bulevirtide	SC	NTCP receptor inhibition	EMA-approved 2 mg qd	2–10 mg qd; open-ended therapy	No dose-dependent differences in CR (~50% at week 48–144) or sustained HDV RNA negativity at post-treatment week 48; continued treatment needed for improved outcomes; delayed treatment linked to worse outcomes; better responses seen with lower baseline HDV RNA and HBsAg, on-treatment HBsAg loss or ≥1 log IU/mL decline, or ADA development; outcomes similar between patients with and without compensated cirrhosis.
	Bulevirtide + PegIFN-α		NTCP receptor inhibition + immune activation	Phase 2 (MYR203, MYR204, SEE-D)	2–10 mg qd + PegIFN-α × 96 wk	Undetectable HDV RNA at post-treatment week 48 in ~25–45%; higher efficacy observed with combination therapy than pegIFN-α or bulevirtide monotherapy, but with increased pegIFN-α-related side effects
Viral assembly inhibition	Lonafarnib + Ritonavir	PO	Inhibition L-HDAg prenylation → virion assembly	Phase 3 (D-LIVR)	50 mg BID + Ritonavir × 48 wk	VR 14.6%, BR 24.7%, histologic improvement 33%; GI intolerance is dose-limiting; better bioavailability and tolerability with ritonavir (CYP3A4 inhibitor); compared to pegIFN-α monotherapy, better biochemical response but lower virologic and histologic outcomes
	Lonafarnib + Ritonavir + PegIFN-α		Inhibition L-HDAg prenylation + immune activation		50 mg BID + Ritonavir + PegIFN-α × 48 wk	VR 32%, BR 34.4%, histologic improvement 53%; triple regimen shows superior efficacy across all outcomes compared to dual therapy, but with increased pegIFN-α-related side effects
Nucleic-acid–directed therapies	Elebsiran (VIR-2218) + Tobevibart (VIR-3434)	SC	siRNA; silences HBV mRNA → ↓ HBsAg + mAb against HBsAg → HBsAg neutralisation	Phase 2 (SOLSTICE) → Phase 3 planned to commence in 2025	200 mg q4wk + Tobevibart × 24–60 wk	100% VR at week 24; HDV RNA undetectable 41% at week 24, 64% at week 36, ≈80% at week 60
	JNJ-3989 (JNJ-73763989)	SC	siRNA; designed to silence all HBV RNA transcription and accordingly translation of HBV viral proteins	Phase 2 (REEF-D)	100 mg q4wk + NA × 144 wk (results announced until wk 48)	At week 48, VR 52%, BR 41%; increased risk of ALT flares in patients with baseline HBsAg levels > 10,000 IU/mL; higher baseline levels of HBsAg and HDV RNA were associated with worse outcomes
	REP 2139-Ca/-Mg	IV/SC	NAP; binds host chaperones → blocking assembly and secretion of HBsAg	Phase 2 (REP 301, REP 301-LFT, RCAP ^‡^)	REP 2139-Ca: 500 mg IV qwk × 15 wk → 250 mg IV qwk + PegIFN-α × 15 wk → PegIFN-α × 33 wk (total 63 wk) REP 2139-Mg: 250 mg IV qwk ± PegIFN-α × 48 wk	REP 301: HDV RNA undetectable in 11/12 at end of therapy, 7/12 at 1 year post-treatment and 7/11 at 3.5 year post-treatment RCAP ^‡^: 76% VR and 61% HDV RNA undetectable at week 48; similar efficacy with/without pegIFN-α.
Antibody-based therapies—Monoclonal	Brelovitug (BJT-778)	SC	Anti-HBsAg IgG1 → HBsAg neutralisation	Phase 2 ongoing; FDA BTD, EMA PRIME/Orphan; Phase 2b/3 (AZURE-1) enrolling	300–600 mg qwk × 48 wk	100% VR with 60% undetectable HDV RNA at week 28 with 300 mg weekly dosing; better results with 600 mg weekly dosing at week 12 with no available data for week 28
	Tobevibart (VIR-3434) ± Elebsiran (VIR-2218)	SC	Anti-HBsAg IgG1 → HBsAg neutralization + silencing of HBV mRNA	Phase 2 (SOLSTICE)	300 mg q4wk + Elebsiran or 300 mg q2wk × 24–60 wk	Better results with combination therapy than monotherapy; see Elebsiran + Tobevibart
	RG-6449 (RO7565020), 2H5-A14, E6F6, N6HB426-20, CIM212930 (early mAbs)		Anti-HBsAg (RG-6449); preS1-NTCP block (2H5-A14); Fc-mediated HBsAg clearance (E6F6); direct NTCP block (N6HB426-20, CIM212930)	Phase 1/pre-clinical	—	—
Antibody-based therapies—Polyclonal	GIGA-2339	IV	>1000 anti-HBsAg clones—broad neutralization	Phase 1 HBV; HDV pre-clinical	—	Potent in-vitro HDV neutralization

Doses and response rates reflect selected trial data and may vary across studies. ^†^ Most studied protocol doses and durations. ^‡^ All patients had prior virologic or biochemical failure on pegIFN-α and/or bulevirtide. Abbreviations (in alphabetical order): ADA = anti drug antibody; ALT = alanine aminotransferase; BID = twice daily; BR = biochemical response; BTD = Breakthrough Therapy designation; CR = combined response (virologic + biochemical); EMA = European Medicines Agency; EVR = early virologic response; FDA = U.S. Food and Drug Administration; HBV = hepatitis B virus; HBsAg = hepatitis B surface antigen; HCV = hepatitis C virus; HDV = hepatitis D virus; IFN = interferon; IV = intravenous; L HDAg = large hepatitis D antigen; mAb = monoclonal antibody; NA = nucleos(t)ide analogue; NAP = nucleic acid polymer; NTCP = sodium taurocholate co transporting polypeptide; PO = oral; PRIME = Priority Medicines designation (EMA); qd = once daily; qwk = once weekly; q2wk = every two weeks; q4wk = every four weeks; SC = subcutaneous; siRNA = small interfering RNA; SVR = sustained virologic response; VR = virologic response (≥2 log HDV RNA decline).

## Data Availability

No new data were created or analyzed in this study. Data sharing is not applicable to this article.

## Abbreviations

Antisense oligonucleotides (ASOs); chronic HDV infection (CHD); early virologic response (EVR); European Medicines Agency (EMA); Food and Drug Administration (FDA); hepatitis B immunoglobulin (HBIG); hepatitis B virus (HBV); hepatitis B virus surface antigen (HBsAg); hepatitis D antigen (HDAg); hepatocellular carcinoma (HCC); hepatitis delta virus (HDV); large HDAg (L-HDAg); melanoma differentiation antigen 5 (MDA5); normalization of alanine aminotransferase (ALT); nucleic acid polymers (NAPs); nucleos(t)ide analogues (NA); pegylated interferon alfa (pegIFN-α); pegylated interferon-λ (pegIFN-λ); RNA-induced silencing complex (RISC); Replicor Compassionate Access Program (RCAP); small HDAg (S-HDAg); small interfering RNAs (siRNAs); sodium taurocholate cotransporting polypeptide (NTCP); sustained virologic response (SVR).
